# Kidney injury molecule-1 expression in human kidney transplants with interstitial fibrosis and tubular atrophy

**DOI:** 10.1186/s12882-015-0011-y

**Published:** 2015-02-13

**Authors:** Aline Lima Nogare, Francisco Veríssimo Veronese, Virna Nowotny Carpio, Rosangela Munhoz Montenegro, José Alberto Pedroso, Karla Laís Pegas, Luiz Felipe Gonçalves, Roberto Ceratti Manfro

**Affiliations:** Post-Graduate Medical Sciences Program, School of Medicine, Federal University of Rio Grande do Sul, Porto Alegre, RS Brazil; Renal Transplant Unit, Division of Nephrology, Hospital de Clínicas de Porto Alegre, Post-Graduate Medical Sciences Program, School of Medicine, Federal University of Rio Grande do Sul 2350, Ramiro Barcelos St. Room 2030, Zip Code: 90035-903 Porto Alegre, RS Brazil; Division of Pathology, Hospital de Clínicas de Porto Alegre, Porto Alegre, RS Brazil

**Keywords:** Kidney transplantation, Fibrosis, Interstitial fibrosis and tubular atrophy, Kidney injury molecule-1

## Abstract

**Background:**

Kidney injury molecule-1 (KIM-1) is expressed in tubular epithelial cells after injury and may have a role in the development of renal graft fibrosis. In this study we evaluated the molecular and protein expressions of KIM-1 in dysfunctional allografts and also mRNA KIM-1 expression in urine as potential biomarkers of graft fibrosis.

**Methods:**

Protein and mRNA levels in renal tissue and urinary sediment cells of 69 kidney transplant recipients that undertook for-cause graft biopsies were evaluated by immunohistochemistry and real-time polymerase chain reaction. The histopathology was classified according to the 2007 Banff schema.

**Results:**

KIM-1 protein expression was increased in biopsies with interstitial fibrosis and tubular atrophy (IF/TA) compared with biopsies showing acute calcineurin inhibitor nephrotoxicity (CIN) (P <0.05). Kidney tissue KIM-1 mRNA signaling (in) was increased in biopsies with IF/TA compared with all other groups (P <0.05). In the urine cells KIM-1 mRNA was also increased in patients with IF/TA compared with patients with acute CIN (P <0.05). Significant correlations were found between KIM-1 protein and mRNA levels in tissue, between mRNA expressions in tissue and urine and between protein tissue expression and gene expression in the urine.

**Conclusions:**

KIM-1 seems to be a marker of kidney graft fibrosis. Urinary KIM-1 mRNA may become a useful non-invasive biomarker of the injuries that can trigger intra-graft fibrotic processes, such as interstitial fibrosis and tubular atrophy.

## Background

In the last decades renal transplantation has become the therapy of choice for many patients with end-stage renal disease. Compared with chronic dialysis, kidney transplantation offers longer survival and better quality of life [1]. However, a variety of injuries to the allograft shortens significantly transplant survival as demonstrated by currently observed half-lives, still much inferior then desired [2]. These injuries lead to allograft fibrosis that is mainly caused by continuous alloimmune responses, drug toxicities and viral infections [3]. Also, when the mechanism leading to fibrosis can not be identified by histological examination, the denomination interstitial fibrosis and tubular atrophy is used in accordance with the Banff classification [4]. Usually the fibrosis driving process occurs silently and is not perceived until the allograft starts to loose function or a surveillance renal biopsy is performed.

KIM-1 (kidney injury molecule-1) is a type 1 transmembrane glycoprotein not detectable in normal kidney tissue but inducible by ischemic and toxic insults. This protein expresses in elevated amounts in the apical surface of surviving proximal tubule epithelial cells and is detectable in the urine [5]. Currently, the link between acute injuries and fibrosis is poorly understood. Peritubular capillary loss, a known consequence of acute injury, is proposed to lead to chronic hypoxia followed by the development of tubulointerstitial fibrosis [6]. It is conceivable that clinical or subclinical aggressions to the renal graft may trigger pro-fibrotic mechanisms which lead to tissue fibrosis, and ultimately to loss of graft function. In our previous studies we found that KIM-1 messenger RNA, along with another fibrosis related molecules, to be augmented in renal allografts with fibrosis [7,8].

In order to study this molecule as a non-invasive biomarker of undergoing fibrosis in renal grafts we evaluated the molecular and protein expressions of KIM-1 in the renal tissue of kidney allograft recipients and also quantified its mRNA in urinary sediment cells (USC).

## Methods

### Patients

Sixty-nine kidney transplant recipients submitted to 81 indication graft biopsies were included. In all patients immunosuppressive therapy was initiated before transplantation and consisted of corticosteroids, sodium mycophenolate and calcineurin inhibitors. Twenty-four patients received induction therapy with Basiliximab® and six with Thymoglobulin®. Immunosuppressive therapy was kept throughout the course of the transplant and adjusted overtime according to our protocol, blood levels of immunosuppressive drugs and the occurrence of acute rejection, drug toxicities, infections and side effects. Graft dysfunction was investigated for vascular, urological, drug toxicity or infectious causes by Doppler ultrasound, nuclear scans, calcineurin inhibitors blood levels and urine cultures before the biopsies were performed. For the biopsies two cores were obtained under ultrasound guidance, using a 16G semi-automatic biopsy gun. One third of the second obtained core was immediately frozen in liquid nitrogen and maintained at −70°C for later RNA extraction. Slide evaluation was performed by a kidney transplant pathologist unaware of the clinical data. The Banff-2007 classification was used for the histopathological diagnoses [4]. The final diagnosis for each biopsy situation was established by evaluation of the pathological criteria, clinical course, response to treatments and outcomes. Patients and biopsies were then classified into four major diagnostic groups: (a) acute tubular necrosis (ATN; n = 9), acute rejection (AR; n = 43), acute calcineurin inhibitor nephrotoxicity (CIN; n = 9) and interstitial fibrosis and tubular atrophy (IF/TA; n = 20). Six protocol biopsies obtained in stable recipients and interpreted as normal kidney graft biopsies were used as a calibrator for the molecular analyzes. Urine was collected in a sterile recipient immediately before each biopsy, centrifuged and processed as described below.

All patients agreed to participate in the study and signed an informed consent form. This study was approved by the Research Ethics Committee of Hospital de Clínicas de Porto Alegre, which is accredited by the National Research Council of the Brazilian Ministry of Health and registered at the Office for Human Research Protection- OHRPUSDHHS (Institutional Review Board - IRB 00000921). The study protocol conformed with the ethical guidelines of the 1975 Helsinki Declaration.

### Gene expression analyses

RNA extraction from graft tissue and USC were processed using the QIAamp® RNA Blood mini kit (QIAGEN Inc., Chatsworth, CA, USA), according to the manufacturer’s instructions. Total RNA quantifications were accessed by the Nano’drop® 1000 Spectrophotometer v.3.7 (Thermo Fischer Scientific, Wilmington, DE, USA) and RNA purity was evaluated by the ratio of absorbances at the wave lengths 260/280 nM. Total RNA was reverse transcribed to cDNA using the cDNA High Capacity Kit (Applied Biosystems, Foster City, CA, USA), according to manufacturer’s instructions, to a final volume of 20 μL and stored at −20°C.

Real-time polymerase chain reaction (RT-PCR) was performed in the cDNA from tissue and urine samples by using the TaqMan Universal PCR Master Mix, composed by AmpliTaq Gold® DNA polymerase, Amperase UNG, passive reference (ROX), buffer and dNTP’s (Applied Biosystems, Foster City, CA, USA) and specific primers for KIM-1 mRNA amplification [Applied Biosystems USA (kidney injury molecule-1 - HAVCR1) ID: Hs00273334_m1] according to the manufacturer’s instructions. 18 s rRNA (TaqMan® PDAR) was used as an endogenous control for sample normalization. FAM (6- carboxyfluorescein) and VIC dye-labeled TaqMan minor groove binder (MGB) probes sets were used respectively for HAVCR and 18 s rRNA, as fluorescent reporter dyes and conjugated at 5’ ends of probes to detect amplification products. The amount of FAM or VIC fluorescence in each reaction liberated by the exonuclease degradation of the TaqMan probe during PCR amplification was measured as a function of PCR cycle using ABI 7000 Prism (Applied Biosystems, Foster City, CA). RT-PCR was carried out in duplicates in 96- well plates on 2 μL of cDNA. Thermal cycling conditions were 50°C for 2 minutes, 60°C for 30 minutes followed by heating to 95°C for 5 minutes and 40 cycles using the temperatures of 94°C for 20 seconds and 62°C for 60 seconds. Data were collected by using the ABI PRISM 7000 SDS analytical thermal cycler (Applied Biosystems, Foster City, CA, USA). The relative quantification of target gene expression was performed using the 2^–ΔΔCT^ comparative method where CT (threshold cycle) value is defined as the point where a statistically significant increase in the fluorescence has occurred.

### Immunohistochemistry for KIM-1

Immunohistochemistry was performed on formalin-fixed, paraffin-embedded tissue. Sections were heated in an oven for 30 min at 60°C, deparaffinized in xylene, rehydrated in absolute and 95% ethanol, incubated for 5 min in 4.5% H_2_O_2_ in methanol to block endogenous peroxidase. Antigen were retrieved with Antigen Decloaker™ pH 6.0 (Biocare Medical, Walnut Creek, CA, USA) in a pressure cooker, and blocked with 5% skim milk in PBS for 20 min. Anti-HAVCR1/KIM-1 polyclonal antibody (LS-B2463, LifeSpan BioSciences, Seattle, WA, USA) was diluted at 1:600 and incubated overnight at 4°C. After biotin blockade, slides were incubated with Universal Dako LSAB® + Kit, with streptavidin-horseradish peroxidase (Dako, Carpinteria, CA, USA) for 20 min, and developed with 3.3-diaminobenzidine. All steps included washing with PBS. Slides were counterstained with Harris hematoxylin (Merck, Darmstadt, Germany) and placed in permanent non-aqueous medium (Vector Laboratories, Burlingame, CA, USA). KIM-1 positivity was defined as a brown staining of the non-atrophic proximal tubules according to a semi-quantitative scale: 0 to 3+ (0, no staining; 1+, weak but entire luminal granular staining; 2+, entire luminal moderate granular staining; 3+, entire luminal strong large granular staining [9].

### Statistical analyzes

Data are presented as descriptive analyses, medians and percentiles (P25-75) values. Fisher’s exact test was used to test associations between categorical variables. Nonparametric data was analyzed by Kruskal-Wallis test to compare medians between groups. The correlations between the levels of gene and protein expression of KIM-1 in the biopsies were calculated using the Spearman’s correlation test. The dot-plot representation graphics show the medians of the relative quantification of gene expression. All analyzes were performed using the SPSS (Statistical Package for the Social Sciences) program (version 17.0, Chicago, IL). The statistical significance level was established at P level lower than 0.05.

## Results

Patients and transplant demographics are presented in Table 1. No significant differences were observed among groups in the parameters of age, gender, ethnicity, use of antibody induction therapy, donor type, donor age, and cold ischemia time. Forty-eight (59%) patients received grafts from deceased donors. A significantly higher serum creatinine was observed in the ATN group as compared to the patients classified in the other groups (P <0.05). The AR group also presented higher serum creatinine as compared to the CIN group (P <0.05). Time interval between the transplant surgery and graft biopsy was higher in the IF/TA group as compared to the ATN and AR groups (P <0.05).

**Table 1:** **Demographics and transplant data**

**Pathologic diagnosis**	**ATN**	**AR**	**CIN**	**IF/TA**	**P**
Number of biopsies	9	43	9	20	
Age (years)	41 (40–56)^1^	41 (28–51)	36 (33–54)	39 (30–49)	0.942
Gender (male/female)	4/5	15/28	3/6	10/10	0.644
Ethnicity (caucasian/non-caucasian)	7/2	39/4	8/1	18/2	0.495
Donor (deceased/living)	8/1	26/17	5/4	14/6	0.144
Donor age (years)	61 (42–63)	47 (41–51)	47 (33–68)	52 (48–52)	0.248
Induction (Basiliximab/Thymoglobulin)	2/2	11/3	3/0	8/1	0.573
Serum creatinine^2,3^ (mg/dL)	7.3 (5.1-8.0)	4.3 (2.4-5.9)	1.9 (1.7-2.3)	3.3 (2.3-4.5)	<0.05
Cold ischemia time (hr)	21 (20–26)	16 (11–22)	19 (12–21)	16 (13–18)	<0.05
Time to biopsy^4^ (days)	27 (14–84)	13 (9–49)	31 (18–226)	569 (227–1223)	<0.05

ATN = acute tubular necrosis; AR = acute rejection; CIN = calcineurin inhibitor nephrotoxicity; IF/TA = interstitial fibrosis and tubular atrophy.

^1^Median and interquartile range; ^2^At biopsy; ^3^Kruskall Wallis (AR > CIN and ATN > AR and IF/TA); ^4^Kruskall Wallis (IF/TA > ATN and AR).

**Figure 1:**
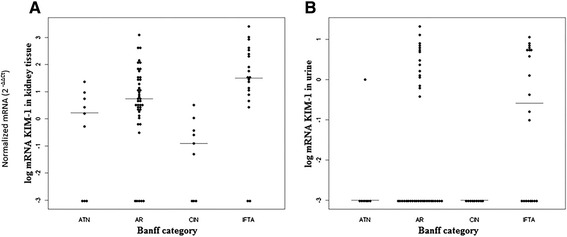
**The dot-plot representation graphics showing the medians and distribution of the quantification levels of normalized KIM-1 mRNA (2** ^**-ΔΔCT**^ **) according to the Banff diagnostic groups.** Panel **A**: kidney tissue. Panel **B**: urinary sediment cells. ATN = acute tubular necrosis; AR = acute rejection; CIN = calcineurin inhibitor-induced nephrotoxicity; IFTA = interstitial fibrosis and tubular atrophy.

Tissue protein expression, measured semi-quantitatively, presented a higher value in the biopsies with a pathological diagnosis of IF/TA. The median KIM-1 protein expression in the IF/TA group [1.5 (1.0-2.0)] was significantly higher than the expression in the ATN [(1.0 (0.0-1.50)], AR [(1.0 (1.0-2.0)] or acute CIN group [0 (0.0 -0.0)] (P = 0.012). Biopsy staining scores of 2+ and 3+ were present in 50% of the grafts with IF/TA, compared to 11%, 22% and 25% of those with CIN, ATN and AR respectively. Tissue mRNA expression was also found to be significantly increased in the kidney biopsies classified in the IF/TA group as compared to all other groups (P <0.05), as shown in Figure 1A. Expression of the mRNA obtained from the cells of the urinary sediment provided a much less intense signal of expression. However, its expression was again significantly higher in the IF/TA group as compared with the acute CNI group (P <0.05) (Figure 1B). Importantly, it was found that a more intense protein expression is paralleled by a higher intensity of the mRNA signal, both in kidney tissue and USC (Table 2), and also that KIM-1 mRNA expression both in tissue and USC increased from IF/TA category I to III (Table 3).

**Table 2:** **Median and percentiles values (P25–P75) of KIM-1 mRNA signaling intensity in kidney tissue and urinary sediment cells according to scores of tissue KIM-1 protein expression**

**KIM-1 protein tissue**	**Tissue KIM-1 mRNA**	**Urinary KIM-1 mRNA**
0	0.02 (0–1.0)	0 (0 – 0)
1+	4.3 (1.41 – 21.7)	0 (0 – 3.2)
2+	32.7 (8.45 – 142.4)	0.6 (0 – 3.4)
3+	1720.9 (885.3 – 1917.4)	5.4 (0 – 8.1)

**Table 3:** **Median and percentiles values (P25–P75) of KIM-1 protein expression in the renal tissue and mRNA expression levels in kidney tissue and urinary sediment cells according to histological IF/TA category**

**IF/TA category**	**Protein expression**	**mRNA tissue expression**	**mRNA urinary cells**
I	1 (1 – 1)	7.6 (2.2 – 206.7)	1.3 (0.1 – 5.6)
II	1.5 (1 – 2)	29.1 (16.1 – 678.4)	0.1 (0.1 – 3.9)
III	2 (1 – 2)	203.7 (32.0 – 324.0)	5.8 (0.2 – 8.4)

Significant correlations were found between protein and mRNA expressions in tissue (*r* = 0.571; P <0.01) and this correlation was stronger in the IF/TA group (*r* = 0.681; P <0.01). A significant correlation was also found between protein expression in tissue and mRNA expression in urinary sediment cells (*r* = 0.318; P <0.01). The correlation between mRNA expression in tissue and urinary cell sediment was also significant (*r* = 0.348; P <0.01).

**Figure 2:**
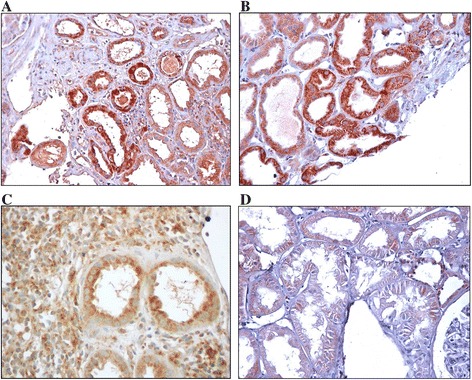
**Immunohistochemical staining of KIM-1 in non-atrophic proximal tubules of kidney transplant for-cause biopsies according to staining intensity.** Slide **A**: interstitial fibrosis and tubular atrophy, 3+; Slide **B**: acute tubular necrosis, 2+; Slide **C**; acute rejection, 1+; Slide **D**: calcineurin inhibitor nephrotoxicity, very weak staining (original magnification x 400 in **A-D**).

Immunohistochemistry slides showing KIM-1 protein expression in patients with post-transplant IF/TA, ATN, AR and CIN are shown in the four panels of Figure 2.

No correlations were observed between donor age and tissue protein expression (*r* = −0.020; P = 0.869), tissue mRNA expression (*r* = −0.023; P = 0.867) and urinary cells mRNA (*r* = −0,095; P = 0.440). Also, an inverse non-significant correlation was observed between cold ischemia time and KIM-1 tissue protein (*r* = −0.183; P = 0.228), tissue mRNA (*r* = −0.083; P = 0.622) and urinary cells mRNA (*r* = −0.188; P = 0.216).

## Discussion

KIM-1 molecule is a type 1 trans-membrane glycoprotein with an intracellular immunoglobulin domain and a highly glycated mucin extracellular portion [10,11]. The cytoplasmic domain is short and has a potentially phosphorilated site indicating that it might have a role in molecular signaling. The ectodomain is clived by a metalloproteinase and the remaining peptide can be detected in the urine [12]. It is also known as TIM-1 (T-cell immunoglobulin mucin-1) and HAVCR-1 (hepatitis A virus cellular receptor-1) [13]. KIM-1 gene is located at the 5q33.2 human chromosome and its expression is undetectable in healthy kidneys or in normal urine [14]. However, its expression increases significantly upon ischemic or toxic injuries, as in acute renal injury, possibly due to a protective role in early lesions, and is also increased in chronic renal failure [5,15].

In the present study we found that, in the renal tissue of for cause kidney allograft biopsies, mRNA expression is paralleled by protein expression and also that USC KIM-1 mRNA transcripts increases in parallel with protein expression. This represents an increment in the information provided in our previous reports in which only the molecular analyzes were performed [7,8]. Molecular and protein expressions were more intense in grafts with the pathological diagnosis of IF/TA suggesting that this molecule may become a feasible biomarker of kidney graft fibrosis.

We have previously shown that KIM-1, along with other fibrosis related genes, have increased mRNA expression of human kidney grafts undergoing IF/TA [7,16]. However, as IF/TA does not precisely identifies the etiology of fibrosis it is probable that among the biopsies classified within the IF/TA group there are cases of chronic calcineurin inhibitor toxicity, chronic alloimune injury and advanced polioma virus nephropathy. In this scenario we believe that KIM-1 is overexpressed in grafts undergoing fibrosis due to different causes. Donor age could be a confounding variable in evaluating KIM-1 expression. In the present work donor age was not significantly different between diagnostic groups and we did not find protein or molecular KIM-1 expressions to be correlated with donor age. Other donor related variables, such as living compared to deceased donors, were not possible to evaluate due to the restricted number of living donor transplants in our series. We also found a considerable variability in the mRNA expressions across the diagnostic groups. It is possible that refinements in the diagnosis of the different cause of fibrosis may provide more reproducible results.

Experimentally, increased production of Kim-1 has been demonstrated predominantly in the S3 portion of the proximal tubule in response to a variety of injuries or as a result of dedifferentiation of the tubular epithelia [17,18]. In native kidneys with fibrotic processes the increased expression of KIM-1 was recently reviewed by Lim et al. [19]. It has being demonstrated that this molecule is significantly expressed in the apical site of dilated tubules of the fibrotic areas in a variety of renal disorders and that KIM-1 expression was associated to the glomerular macrophage influx. Histochemistry double immunostaining studies revealed that KIM-1 is present in these macrophages infiltrating areas, accompanied by fibrosis and dedifferentiated tubular cells [19]. In an elegant set of experiments, Humphreys et al. [20] proposed a novel role for chronic KIM-1 expression in the pathogenesis of renal fibrosis through activation of the innate immune system and leukocyte recruitment. They suggested that persistent KIM-1 expression is maladaptive through chronic uptake of cell toxic components of the tubular lumen, promoting chronic inflammation and ultimately renal fibrosis, making KIM-1 a potential novel therapeutic target in fibrotic kidney diseases.

In humans it has being shown that KIM-1 is expressed predominantly in the proximal apical membrane of necrotic and apoptotic cells of the tubular lumen [21]. Augmented KIM-1 expression was initially described in biopsies samples of patients with ATN were it was also found to be increased in the urine as measured by ELISA [11]. Latter it was found, in a variety of renal diseases, that KIM-1 was primarily expressed at the luminal side of dedifferentiated proximal tubules, in areas with fibrosis and inflammation [22]. Also in humans KIM-1 expression has being found to be increased in other clinical settings such as urate nephropathy and renal cell carcinoma [19].

KIM-1 has not been extensively studied in human renal transplantation [23]. In the early studies by van Timmeren et al. [24] it was found that urinary KIM-1 is an independent predictor of long-term graft loss and it has also being reported, by Jin et al. [25], as predictive of the development of later acute rejection when measured in the blood at day 1 post-transplantation. Zhang et al. [9] reported that KIM-1 tissue staining is a sensitive and specific marker of early proximal tubular injury and correlates with the degree of renal dysfunction. Additionally, KIM-1 mRNA obtained from kidney grafts before implantation correlates inversely with kidney function at procurement and directly with the degree of interstitial fibrosis [26]. Finally, KIM-1 mRNA measurement in the urine has been shown to be predictive of declining renal function and late graft loss independently of acute rejection [27]. Taken together these reports are supportive of our present data showing, as reported in other settings, that in renal transplantation KIM-1 is involved in graft fibrosis processes.

## Conclusions

In the present study the gene expression of KIM-1 was paralleled by protein expression in the renal tissue of for cause kidney allograft biopsies. Messenger RNA transcripts of KIM-1 in urine also increased in parallel with protein expression. Interestingly, molecular and protein expressions were higher in grafts with interstitial fibrosis and tubular atrophy suggesting that KIM-1 represents a potential biomarker of injuries that can lead to kidney allograft fibrosis.

Currently the pathological diagnosis is the gold-standard for the evaluation of the aggressions to the kidney allograft. However, it is highly recognized that there is a need for the development of accurate non-invasive biomarkers to uncover, prognosticate and guide therapy without the need of invasive and risky methods. Messenger RNA profiling in the urine might have the advantage of detecting injury before the initiation of scarring. We believe that improvements on mRNA biomarker research, perhaps along with other platforms, will be the way for providing earlier and better interventions and thus leading to improvement in renal graft survival.

## Abbreviations

ATN: Acute tubular necrosis
AR: Acute rejection
CIN: Calcineurin inhibitor nephrotoxicity
IF/TA: Intersticial fibrosis and tubular atrophy
KIM-1: Kidney injury molecule-1
USC: Urinary sediment cells
